# Differentiation of multiple brain metastases and glioblastoma with multiple foci using MRI criteria

**DOI:** 10.1186/s12880-023-01183-3

**Published:** 2024-01-02

**Authors:** Sebastian Johannes Müller, Eya Khadhraoui, Marielle Ernst, Veit Rohde, Bawarjan Schatlo, Vesna Malinova

**Affiliations:** 1 Department of Neuroradiology, University Medical Center, Göttingen, Germany; 2 Neuroradiologische Klinik, Klinikum Stuttgart, Stuttgart, Germany; 3 Department of Neurosurgery, University Medical Center, Göttingen, Germany

**Keywords:** Brain metastases, Multifocal glioblastoma, Brain imaging, Multicentric glioblastoma

## Abstract

**Objective:**

Glioblastoma with multiple foci (mGBM) and multiple brain metastases share several common features on magnetic resonance imaging (MRI). A reliable preoperative diagnosis would be of clinical relevance. The aim of this study was to explore the differences and similarities between mGBM and multiple brain metastases on MRI.

**Methods:**

We performed a retrospective analysis of 50 patients with mGBM and compared them with a cohort of 50 patients with multiple brain metastases (2–10 lesions) histologically confirmed and treated at our department between 2015 and 2020. The following imaging characteristics were analyzed: lesion location, distribution, morphology, (T2-/FLAIR-weighted) connections between the lesions, patterns of contrast agent uptake, apparent diffusion coefficient (ADC)-values within the lesion, the surrounding T2-hyperintensity, and edema distribution.

**Results:**

A total of 210 brain metastases and 181 mGBM lesions were analyzed. An infratentorial localization was found significantly more often in patients with multiple brain metastases compared to mGBM patients (28 vs. 1.5%, *p* < 0.001). A T2-connection between the lesions was detected in 63% of mGBM lesions compared to 1% of brain metastases. Cortical edema was only present in mGBM. Perifocal edema with larger areas of diffusion restriction was detected in 31% of mGBM patients, but not in patients with metastases.

**Conclusion:**

We identified a set of imaging features which improve preoperative diagnosis. The presence of T2-weighted imaging hyperintensity connection between the lesions and cortical edema with varying ADC-values was typical for mGBM.

## Introduction

Glioblastoma (GBM) is a common brain malignancy and carries a poor prognosis despite advances in treatment [1–4]. Patients suffering from GBM with multiple foci at presentation (mGBM) represent a subgroup with even poorer prognosis [5] and account for 22–35% of GBM cases [6, 7]. In 1963, mGBM were classified into multifocal and multicentric GBM depending on the distance between the tumor lesions and on the presence or absence of a connection between them [8]. While multifocal GBM have at least one visible connection on T2-weighted imaging, by definition no such connection can been found in multicentric GBM comprising more distant lesions, and representing only 2–3% of all GBM cases [9]. In the era of modern magnetic resonance imaging (MRI), different (high-resolution T2-/FLAIR-weighted) sequences were established to better distinguish between the two groups [9]. However, the differentiation between tumor-associated edema and tumor microinvasion (i.e. tumor infiltration area of surrounding brain tissue) remains a diagnostic challenge [10]. Furthermore, other cerebral pathologies such as brain metastases may exhibit a similar presentation on imaging, hence, mimicking mGBM, setting a higher demand on neuroradiology to distinguish between these diagnoses on initial imaging. A reliable and early differentiation on imaging would allow a more targeted diagnostic algorithm in these patients and to avoid redundant diagnostics possibly associated with side effects. From a pathophysiological point of view brain metastases and GBM are two different entities requiring distinct treatment approaches concerning the recommendation for performing radical surgery and radio-chemotherapy or radiation/chemotherapy alone[11–13]. While GBM invasion within brain tissue is supposed to primarily occur through a neuronal pathway [14], the spread of brain metastases is expected to be mainly of hematogenic nature, which can affect the lesion presentation on imaging [15]. Hence, there might be specific imaging features pointing out the underlying pathology. The aim of this study was to explore differences on imaging between mGBM and multiple brain metastases and to develop a diagnostic algorithm for differentiation on initial MRI.

## Methods

### Study design

This was a retrospective single-center non-interventional observational study. Institutional review board approval was obtained (“Ethics committee of University Medical Center Göttingen”, Von-Siebold-Str. 3, D-37075 Göttingen, Number 43/4/23; date of approval 04/19/2023). Informed consent was waived because of the retrospective nature of the study and the anonymization of the clinical data.

### Study population

The diagnosis was confirmed in each patient after performing a biopsy/resection of the tumor with subsequent neuropathological examination (the gold standard for diagnosing brain tumors). In addition to the reliable histological diagnosis, the second main inclusion criterion was the availability of complete initial MRI dataset containing a contrast-enhanced T1-weighted sequence, a T2-weighted sequence including fluid inversion recovery (FLAIR) for detecting cystic parts, and a diffusion-weighted imaging (DWI) sequence with apparent diffusion coefficient (ADC) mapping. Exclusion criteria were age < 18 years, cancer of unknown primary, no complete pre-operative “in-house” MRI scan, and poor MR image quality (e.g., caused by movement artifacts).

**Fig. 1:**
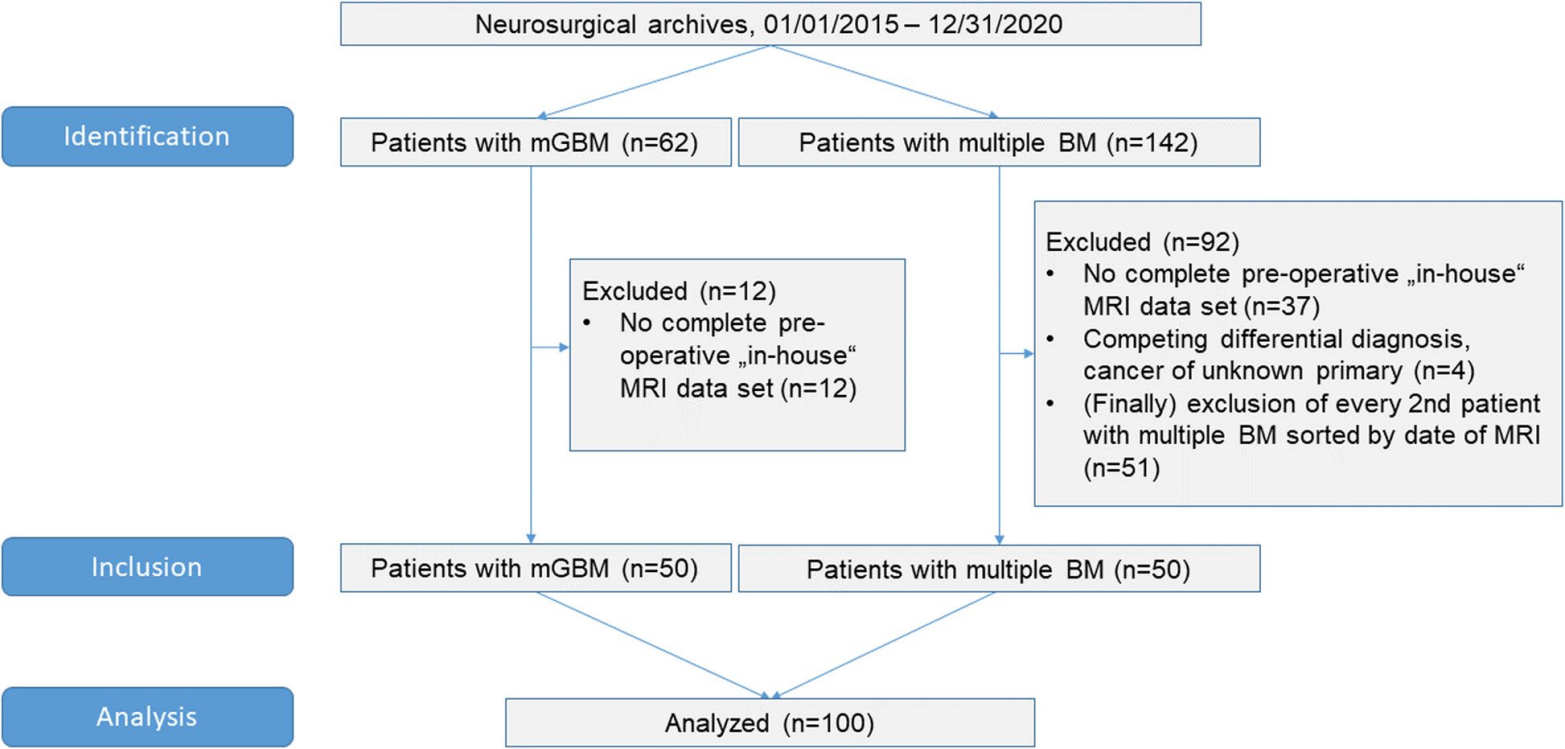
Flow chart of included and analyzed patients. mGBM – glioblastoma with multiple foci; BM – multiple brain metastasis

We identified and included a patient cohort with histologically confirmed GBM presenting on imaging with multiple tumor lesions and a patient cohort with multiple brain metastases (at least two and less than 10 lesions with at least one being resected and histologically confirmed), who were treated at the University Medical Center Göttingen between 01/01/2015 and 12/31/2020. Figure 1 demonstrates a flow chart of included and excluded patients. Patients with singular GBM or singular brain metastasis, and patients with more than 10 lesions were not included. A total of 50 consecutive patients with histologically confirmed GBM presenting with multiple tumor lesions on imaging were identified. With the aim of composing a comparable patient group with multiple brain metastases including the same number of patients treated during the same time-period every second patient was included from a consecutive database of patients with multiple brain metastases, building a group encompassing 50 patients with multiple brain metastases.

### MRI and acquisition parameters

MRI data of two different MR scanners were included (1.5 and 3 Tesla, Siemens MAGNETOM Avanto and Prisma, Siemens AG, Werner-von-Siemens-Str. 1, D-80333 Munich, Germany). Transversal T2 TSE sequences were achieved with a slice thickness of 2.5 mm, as well as transversal T2-/FLAIR sequences with a slice thickness of 4 mm. ADC maps were based on echo-planar imaging diffusion weighted imaging with a slice thickness of 5 mm. Sagittal 3D T1-weighted VIBE sequences with a slice thickness of 1.0 mm were performed before and after contrast agent application (standard dose 0.1 mml/kg, Gadovist®, Bayer AG, Kaiser-Wilhelm-Allee 1, D-51373 Leverkusen, Germany). For more sequence details see Table 1.

**Table 1:** MRI sequence details

MRI	Sequence	TE in ms	TR in ms	TI in ms	Slice thickness in mm	Resolution in mm x mm
1.5 Tesla	3D T1w VIBE sag	3.6	20		1	1 × 1
	T2w TSE tra	110	4700		2.5	0.9 × 0.9
	T2w-FLAIR tra	117	10,000	2600	4	0.5 × 0.5
	EPI DWI/ADC	84	6800		5	0.9 × 0.9
3 Tesla	3D T1w VIBE sag	2.2	5.0		1	1 × 1
	T2w TSE tra	108	3000		2.5	0.5 × 0.5
	T2w-FLAIR tra	96	9000	2500	4	0.5 × 0.5
	EPI DWI/ADC	67	3000		5.2	0.6 × 0.6

*TE* Echo time, *TR* Repetition time, *TI* Inversion time, *VIBE* Volumetric interpolated breath-hold examination, *TSE* Turbo spin echo, *FLAIR* Fluid inversion recovery, *EPI DWI*/*ADC* Echo-planar-imaging diffusion weighted imaging / apparent diffusion coefficient

### Imaging analysis

**Fig. 2:**
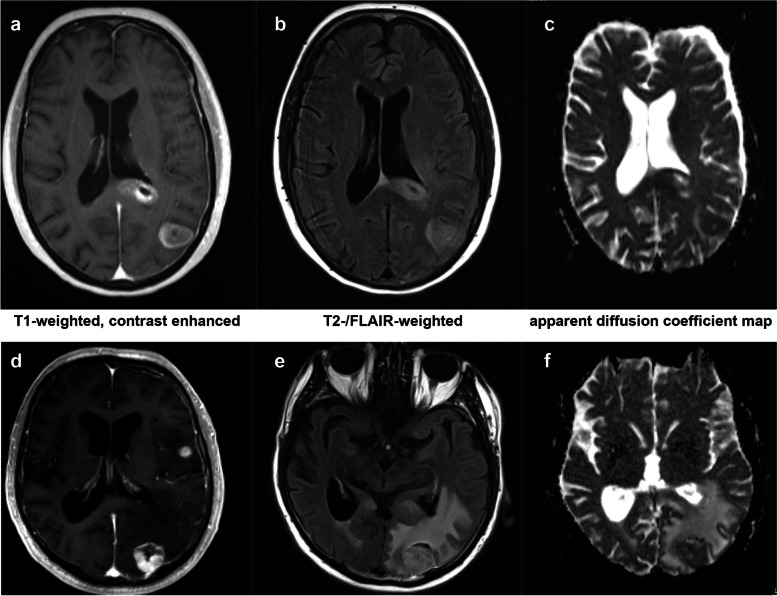
Example of two MRI of the tumor region and edema of a glioblastoma with multiple loci (**a**) – T1-weighted and contrast enhanced; (**b**) – T2/-FLAIR-weighted; (**c**) – ADC map) and of multiple brain metastases (**d**) – T1-weighted and contrast enhanced; (**e**) – T2/-FLAIR-weighted; (**f**) – ADC map). FLAIR – Fluid attenuated inversion recovery; ADC – apparent diffusion coefficient

The imaging analysis included an evaluation of morphology and growth patterns as well as a measurement of T2-/FLAIR-intensity and ADC of tumor lesions and of the surrounding edema. The following radiological parameters were assessed using the contrast-enhanced T1-weighted sequence: 1-size of tumor lesions (mean of the maximal diameters in all three dimensions), 2-regularity of the lesions’ edge (linear, diffuse, mixed), 3-contrast medium uptake, 4-morphology of tumor lesions (cystic, solid, mixed), 5-distance between tumor lesions (shortest distance to next loci measured in contrast-enhanced-T1-weighted 3D-sequences) T2-weighted sequence / FLAIR was used for the evaluation of the following parameters: 1-perilesional edema, 2-connection between tumor lesions, FLAIR-intensity of tumor lesions and of perilesional edema. Examples of MRI of GBM with multiple lesions and multiple brain metastases are demonstrated in Fig. 2.

**Fig. 3:**
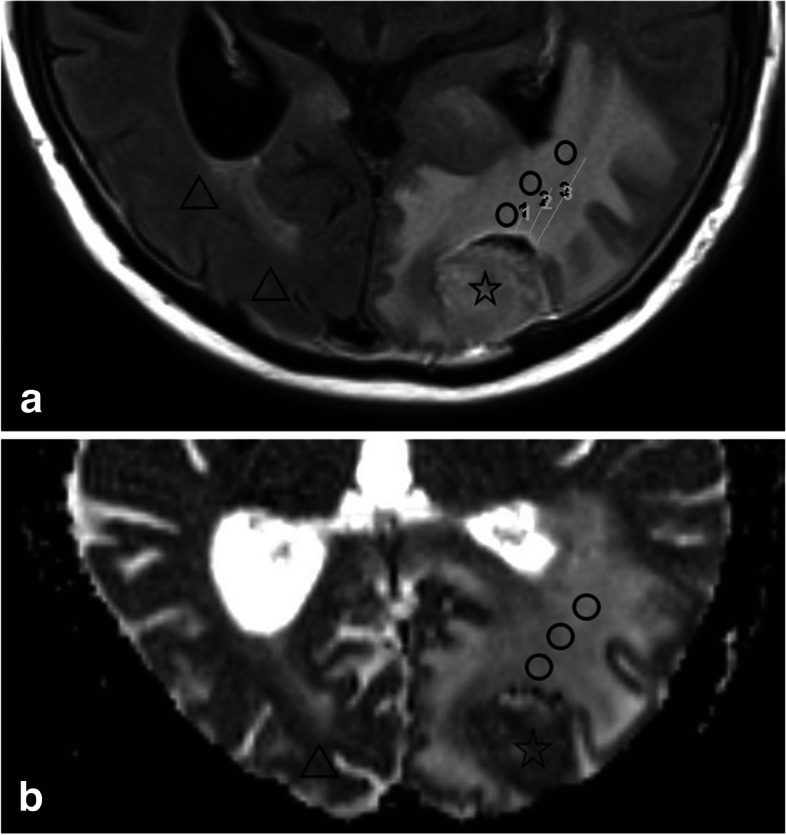
Example of two measurements of the tumor region and edema of multiple brain metastases (**a**) – T2/-FLAIR-weighted; (**b**) – ADC map). STAR—measured tumor region; ADC region, TRIANGLE –reference values of the contralateral healthy white matter; CIRCLES – edema in (1) 5 mm, (2) 10 mm and (3) 20 mm distance to tumor border. FLAIR – Fluid attenuated inversion recovery; ADC – apparent diffusion coefficient

Since both the T2/FLAIR values and the ADC values varied greatly depending on the patient, the specific sequence parameters and the MR scanner, normalization was necessary. For calculation of ADC ratio measurements of the solid tumor and the healthy contralateral white matter were performed as illustrated in Fig. 3. A similar approach was applied for the measurement of edema and for FLAIR- values of tumor and perilesional edema. In order to capture the tumor cell infiltration area surrounding the tumor lesion, we measured the edema at three different distances from the tumor lesion, directly adjacent to the tumor with 5 mm distance, in moderate distance of 10 mm, and in long distance of 20 mm, whenever feasible dependent on the size of the lesion and edema. For comparing ADC values of supra- and infra-tentorial tumor lesions of different field strengths we used a simple procedure as described in previous publications [16–18]. The imaging analysis was performed by two neuroradiology fellows (SM, EK) with four and five years of experience in brain MRI diagnostics respectively, blinded to clinical information. They independently evaluated the cases and measured the FLAIR and ADC values. The software used was GE Centricity™ Universal Viewer (GE Healthcare, 500 W Monroe St, Chicago, IL 60661, United States).

### Statistical analysis

The program Statistica, version 13 (TIBCO Software Inc., Palo Alto, CALIFORNIA, USA) was used. Significance level was set to *p* < 0.05. The comparison of parameter of GBM with multiple tumor lesions and multiple brain metastases was performed using two-sided t-test, respectively binomial test in case of Boolean data. For the comparison of three or more subgroups, analysis of variance (ANOVA) post-hoc (Tukey’s test) [19] was performed using r (https://www.r-project.org/). Receiver Operating Curves (ROC) and Area Under Curve (AUC) values were used for calculating the ADC ratio with the highest diagnostic value to differentiate GBM with multiple tumor lesions from multiple brain metastases. The global optimum was determined by maximizing Youden’s J. The inter-rater agreement was calculated using the intraclass correlation coefficient (ICC) with R (packages irr, readxl, lpSolve and psych) based on mean-rating, absolute-agreement, and a 2-way random-effects model. Effect sizes (Cohen’s d) of t-Tests were calculated and evaluated as described [20].

## Results

### Study population

A total of 50 patients with GBM with multiple tumor lesions and 50 patients with multiple brain metastases (2–10 tumor lesions) were included in the study. Mean age of patients with multiple GBM lesions was 65 ± 13 (mean ± standard deviation, range 36–91) years, 68% of whom (34/50) were male, and 32% (16/50) were female. Patients with multiple brain metastases were on average 66 ± 10 (mean ± standard deviation, range 43–85) years old, 54% (27/50) of whom were male, and 46% (23/50) were female. A summary of baseline characteristics of the patients is given in Table 2.

**Table 2:** Baseline characteristics of study population

Parameter	GBM with multiple tumor lesions	Multiple brain metastases	*p*-value binomial test
**Number of patients**	50	50	
**Mean age ± SD**	65 ± 13	66 ± 10	
**Sex**			
Male (%)	34/50 (68%)	27/50 (54%)	
Female (%)	16/50 (32%)	23/50 (46%)	
**Histology**			
Lung cancer		30/50 (60%)	
Breast cancer		7/50 (14%)	
Melanoma		7/50 (14%)	
Colorectal cancer		2/50 (4%)	
Gastric cancer		1/50 (2%)	
Ovarian cancer		1/50 (2%)	
Transitional cell carcinoma		1/50 (2%)	
Clear cell renal cancer		1/50 (2%)	
**Mean number of tumor lesions ± SD;** **(range) per patient**	3.6 ± 1.5 (2–7)	4.2 ± 2.3 (2–10)	
**Total number of lesions**	181	210	
**Location of tumor lesions**			
Frontal white matter	48/181 (27%)	64/210 (30%)	0.47
Parietal white matter	22/181 (12%)	25/210 (12%)	0.47
Temporal white matter	40/181 (22%)	34/210 (16%)	0.26
Occipital white matter	8/181 (4%)	22/210 (10%)	** < 0.001**
Diencephalon	18/181 (10%)	2/210 (1%)	** < 0.0001**
Brain stem	4/181 (2%)	1/210 (0.5%)	0.09
Cerebellum	1/181 (0.5%)	59/210 (28%)	** < 0.0001**
Corpus callosum	22/181 (12%)	0/210 (0%)	** < 0.0001**
Septum pellucidum	2/181 (1%)	0/210 (0%)	0.11
Basal ganglia	8/181 (4%)	2/210 (1%)	0.12
Insula	4/181 (2%)	0/210 (0%)	**0.03**
Ependym	1/181 (0.5%)	1/210 (0.5%)	0.33
**Total number of lesions**	181	210	

*SD* Standard deviation

### Imaging characteristics of GBM with multiple tumor lesions

The mean number of detected GBM tumor lesions per patient was 3.6 ± 1.5 (range 2–7, median 3). In 24% (12/50) of all GBM cases multicentric tumor lesions were found without visible FLAIR-connection between the single lesions, and in 34% (17/50) multifocal tumor lesions were detected with visible connection between the tumor lesions. In the remaining 42% (21/50) of cases both types were found with tumor lesions without as well as some with visible connection between the tumor lesions. Contrast enhancement was found in 77 ± 28% of multiple GBM loci per patient (range 0–100%, median 100%). In 94% (47/50) of GBM cases the tumor lesions were found to have a supratentorial location. Only 6% (3/50) had an infratentorial location.

While the tumor location within the white matter of each brain lobe was comparable between GBM tumor lesions und multiple brain metastases, involvement of midline structures such as corpus callosum, septum pellucidum were only found in GBM patients. Furthermore, an ependymal location or an involvement of the insula was only seen in GBM patients. The mean measured distance between individual GBM tumor loci was 10.8 ± 14.4 mm (range 0–73 mm, median 5 mm). In patients with multifocal GBM, the mean distance between the lesions was 3.7 ± 7.6 mm (range 0–44 mm, median 0), that was significantly shorter compared to patients with multicentric GBM 25.0 ± 16.9 mm (range 0–73 mm, median 22), t-test *p* < 0.01. The measured distance between tumor lesions in patients with multifocal and multicentric lesions was 11.5 ± 13.5 mm (range 0–62 mm, median 6). The mean measured diameter on contrast-enhanced T1-weighted sequence of GBM tumor lesions was 24.8 ± 16.0 mm (range 2–83 mm, median 22). No significant differences among the subgroups regarding tumor size were detected (multifocal: 27.7 ± 18.2 mm; mixed: 24.0 ± 14.2 mm; multicentric: 22.6 ± 16.2 mm).

The evaluation of the regularity of the lesions’ edge / margin was discarded due to low interrater reliability of the two neuroradiologists (for both mGBM and brain metastasis). The impression of a transition of an edge between diffuse and linear appears to be highly subjective. All GBM tumor lesions were morphologically classified as follows: 65/181 were cystic, 25/181 were solid, 25/181 were mixed with cystic and solid parts, and 66/181 showed a cortical swelling.

The mean measured diameter (FLAIR-hyperintensity) of perilesional edema in GBM patients was 6.6 ± 8.4 mm (range 0–40 mm, median 3). While multifocal GBM tumor lesions had a slightly (but not significantly, t-test; *p* = 0.32) more pronounced edema (8.1 ± 9.8 mm, range 0–40, median 5), the multicentric with 6.0 ± 6.6 mm (range 0–19, median 3) and the mixed group with 5.7 ± 7.7 mm (range 0–30 mm, median 2 mm) showed no differences. In contrast, the tumor-edema-ratio differed in the multicentric subgroup (5.3 ± 4.9, median 3.9) from the mixed (4.0 ± 4.6, median 2.2) and the multifocal group (4.1 ± 3.2, median 2.8). However, Tukey’s Test revealed no significant differences between the GBM subgroups. Results of subgroup (ANOVA post-hoc) analysis regarding FLAIR and ADC ratios are demonstrated in Table 3.

**Table 3:** Results (*p*-values) of Tukey’s test of four groups (multiple BM, multifocal mGBM, multicentric mGBM and the mixed mGBM group) for FLAIR and ADC ratios

Parameter	ANOVA model Pr (> F)	Multifocal mGBM vs multicentric mGBM	Multifocal mGBM vs mixed mGBM	Mixed mGBM vs. multicentric mGBM	Multifocal mGBM vs multiple BM	Multiple BM vs multicentric mGBM	Multiple BM vs mixed mGBM
ADC-ratio (tumor)	0.246	0.855	0.482	0.992	0.999	0.763	0.248
ADC-ratio (edema 5 mm)	**0.00001**	0.991	0.778	0.727	**0.005**	0.160	**0.00001**
ADC-ratio (edema 10 mm)	0.216	0.973	0.979	0.998	0.625	0.597	0.319
ADC-ratio (edema 20 mm)	0.873	0.985	0.980	0.999	0.998	0.949	0.896
FLAIR-ratio (tumor)	**0.00001**	0.993	0.225	0.272	**0.0001**	**0.001**	**0.009**
FLAIR-ratio (edema 5 mm)	0.061	0.121	0.786	0.382	0.999	0.054	0.629
FLAIR-ratio (edema 10 mm)	0.055	0.377	0.960	0.619	0.706	0.075	0.384
FLAIR-ratio (edema 20 mm)	0.078	0.371	0.719	0.839	0.982	0.134	0.270

*mGBM* Glioblastoma with multiple foci at presentation, *BM* Brain metastases, *ADC* Apparent diffusion coefficient, *FLAIR* Fluid attenuated inversion recovery, edemas were measured in 5, 10 and 20 mm distance (if available) from the contrast enhancing tumor. Bold – differs significantly *p* < .05

### Imaging characteristics of multiple brain metastases

The mean number of detected brain metastases loci per patient was 4.2 ± 2.3 (range 2–10, median 4). Contrast enhancement was found in 98 ± 1% of brain metastases loci per patient (range 75–100%, median 100%). In 37 of included cases no FLAIR-connection between the brain metastases could be found, in twelve patients two lesions were connected and only in one patient three connected lesions were detected. Primary tumors were lung cancer (60%), breast cancer (14%), melanoma (14%), colorectal cancer (4%), gastric cancer (2%), ovarian cancer (2%), transitional cell carcinoma (2%) and clear cell renal cell carcinoma (2%). Brain metastases presented in 46% (23/50) in a supratentorial location, 8% (4/50) have an infratentorial location, where the remaining 46% (23/50) had simultaneously supra- and infratentorial lesions. The mean measured distance of multiple brain metastases was 44.2 ± 28.3 mm (range 2–128 mm, median 43 mm), that was significantly longer compared to GBM with multiple tumor lesions (t-test, *p* < 0.01). The mean measured diameter of multiple brain metastases was 14.0 ± 12.8 mm (range 1–60 mm, median 9), that was significantly smaller compared to GBM with multiple tumor lesions (t-test, *p* < 0.01).

In patients with multiple brain metastases 41% (91/210) of lesions were solid, 24% (53/210) of lesions were cystic, 30% (66/210) of lesions were mixed with solid and cystic parts, and no lesions were classified as cortical swelling.

The mean measured diameter of multiple brain metastases associated edemas was 8.7 ± 12.1 mm (range 0–87 mm, median 4). The tumor-edema-ratio was only 3.1 ± 5.2 (median 1.8). The measured ADC values and calculated ratios are demonstrated in Table 4. A significant (*p* < 0.02) higher ADC ratio of multiple brain metastases associated edema was detected adjacent to the tumor.

**Table 4:** ADC values (mean ± standard deviation) and ratios with 95%-confidence interval

**Parameter**	**Multiple brain metastases**	**mGBM**	Multifocal mGBM	Multicentric mGBM	Mixed mGBM
ADC-value (tumor)	673 ± 305	721 ± 323	742 ± 296	796 ± 262	685 ± 352
ADC-value (edema 5 mm)	1512 ± 276*	1227 ± 526	1353 ± 501	1391 ± 322	1066 ± 323*
ADC-value (edema 10 mm)	1593 ± 305	1498 ± 466	1642 ± 329*	1554 ± 387	1343 ± 387*
ADC-value (edema 20 mm)	1653 ± 307	1561 ± 584	1816 ± 307	1684 ± 279	1296 ± 279
ADC-ratio (tumor)	0.97 ± 0.48 (0.87–1.07)	1.05 ± 0.35 (0.99–1.11)	0.98 ± 0.34 (0.88–1.08)	1.06 ± 0.41 (0.89–1.24)	1.09 ± 0.34 (1.01–1.17)
ADC-ratio (edema 5 mm)	2.17 ± 0.41** (2.08–2.25)	1.79 ± 0.54 (1.67–1.91)	1.83 ± 0.52 (1.63–2.03)	1.88 ± 0.55 (1.55–2.21)	1.72 ± 0.56 (1.53–1.91)
ADC-ratio (edema 10 mm)	2.25 ± 0.41 (2.16–2.35)	2.07 ± 0.55 (1.91–2.23)	2.11 ± 0.48 (1.89–2.33)	2.02 ± 0.66 (1.41–2.63)	2.05 ± 0.59 (1.78–2.32)
ADC-ratio (edema 20 mm)	2.36 ± 0.47 (2.23–2.49)	2.28 ± 0.51 (2.07–2.49)	2.34 ± 0.45 (1.99–2.68)	2.24 ± 0.61 (1.48–2.99)	2.25 ± 0.55 (1.88–2.62)

*mGBM* Glioblastoma with multiple foci at presentation, *BM* Brain metastases, *ADC* Apparent diffusion coefficient, edemas were measured in 5, 10 and 20 mm distance (if available) from the contrast enhancing tumor. * -differs significantly *p* < .05 from some other groups with t-Test; ** -differs significantly *p* < .05 from all other groups with t-Test

### Differentiation between GBM with multiple tumor lesions and multiple brain metastases on initial magnetic resonance imaging (decision flow chart)

The AUC in ROC-analysis of mean distance between the lesions for differentiation of mGBM and multiple brain metastases was 0.88, with an optimized threshold of 17 mm (Youden's J, sensitivity 78%, specificity 84%). The AUC in ROC-analysis of mean tumor lesion size for differentiation of mGBM and multiple brain metastases was 0.73, with an optimized threshold of 13 mm (Youden's J, sensitivity 55%, specificity 77%). There was no significant difference of the tumor (t-test, *p* = 0.58) and edema sizes (t-test, *p* = 0.11) as well as the tumor-edema ratio (t-test, *p* = 0.61) between GBM with multiple tumor lesions and multiple brain metastases. Normalized FLAIR ratios of edemas revealed no additional significant information (see Table 3), but the mean FLAIR ratios of multiple brain metastases were significantly (*p* < 0.03) lower than those of GBM with multiple tumor lesions (cystic parts of lesions were not measured).

The effect size of a t-test using the FLAIR ratio (tumor)for differentiation of GBM with multiple tumor lesions from multiple brain metastases was *d* = 0.58 (moderate) with *p* = 0.0001.

The AUC in ROC-analysis of FLAIR ratio for differentiation of GBM with multiple tumor lesions from multiple brain metastases was 0.68, with an optimized threshold of 1.39 (Youden's J, sensitivity 57%, specificity 69%). Measured T2-/FLAIR-mean values are in Table 5.

**Table 5:** FLAIR values (mean ± standard deviation) and ratios with 95% confidence interval

**Parameter**	**Multiple brain metastases**	**mGBM**	Multifocal mGBM	Multicentric mGBM	Mixed mGBM
FLAIR-value (tumor)	418 ± 100**	470 ± 110	495 ± 157	461 ± 79	459 ± 87
FLAIR-value (edema 5 mm)	565 ± 104	535 ± 149	577 ± 225	478 ± 120	533 ± 79*
FLAIR-value (edema 10 mm)	571 ± 98	562 ± 172	583 ± 241*	508 ± 100	562 ± 73*
FLAIR-value (edema 20 mm)	566 ± 100	537 ± 145	531 ± 230	505 ± 113	558 ± 68
FLAIR-ratio (tumor)	1.38 ± 0.34** (1.31–1.43)	1,56 ± 0.29 (1.52–1.61)	1.62 ± 0.31 (1.53–1.71)	1.64 ± 0.28 (1.52–1.76)	1.51 ± 0.28* (1.45–1.57)
FLAIR-ratio (edema 5 mm)	1.87 ± 0.33* (1.81–1.94)	1.80 ± 0.30 (1.73–1.87)	1.88 ± 0.29* (1.75–2.00)	1.64 ± 0.34* (1.45–1.84)	1.80 ± 0.27 (1.71–1.89))
FLAIR-ratio (edema 10 mm)	1.94 ± 0.30 (1.86–2.01)	1.80 ± 0.28 (1.71–1.89)	1.85 ± 0.33 (1.68–2.02)	1.63 ± 0.08 (1.54–1.72)	1.80 ± 0.24 (1.66–1.93)
FLAIR-ratio (edema 20 mm)	1.93 ± 0.29 (1.84–2.02)	1.78 ± 0.27 (1.67–1.89)	1.89 ± 0.41 (1.57–2.21)	1.64 ± 0.06 (1.56–1.71)	1.76 ± 0.15 (1.66–1.86)

*mGBM* Glioblastoma with multiple foci at presentation, *BM* Brain metastases, *FLAIR* Fluid attenuated inversion recovery, edemas were measured in 5, 10 and 20 mm distance (if available) from the contrast enhancing tumor. * -differs significantly *p* < .05 from some other groups with t-Test ** -differs significantly *p* < .05 from all other groups with t-Test

The effect sizes of a t-test using the ADC ratio (5 and 10 mm) for differentiation of both groups (of GBM with multiple tumor lesions and multiple brain metastases) was *d* = 0.81 (*p* = 0.00001; excellent) and *d* = 0.38 (*p* = 0.039; low).

**Fig. 4:**
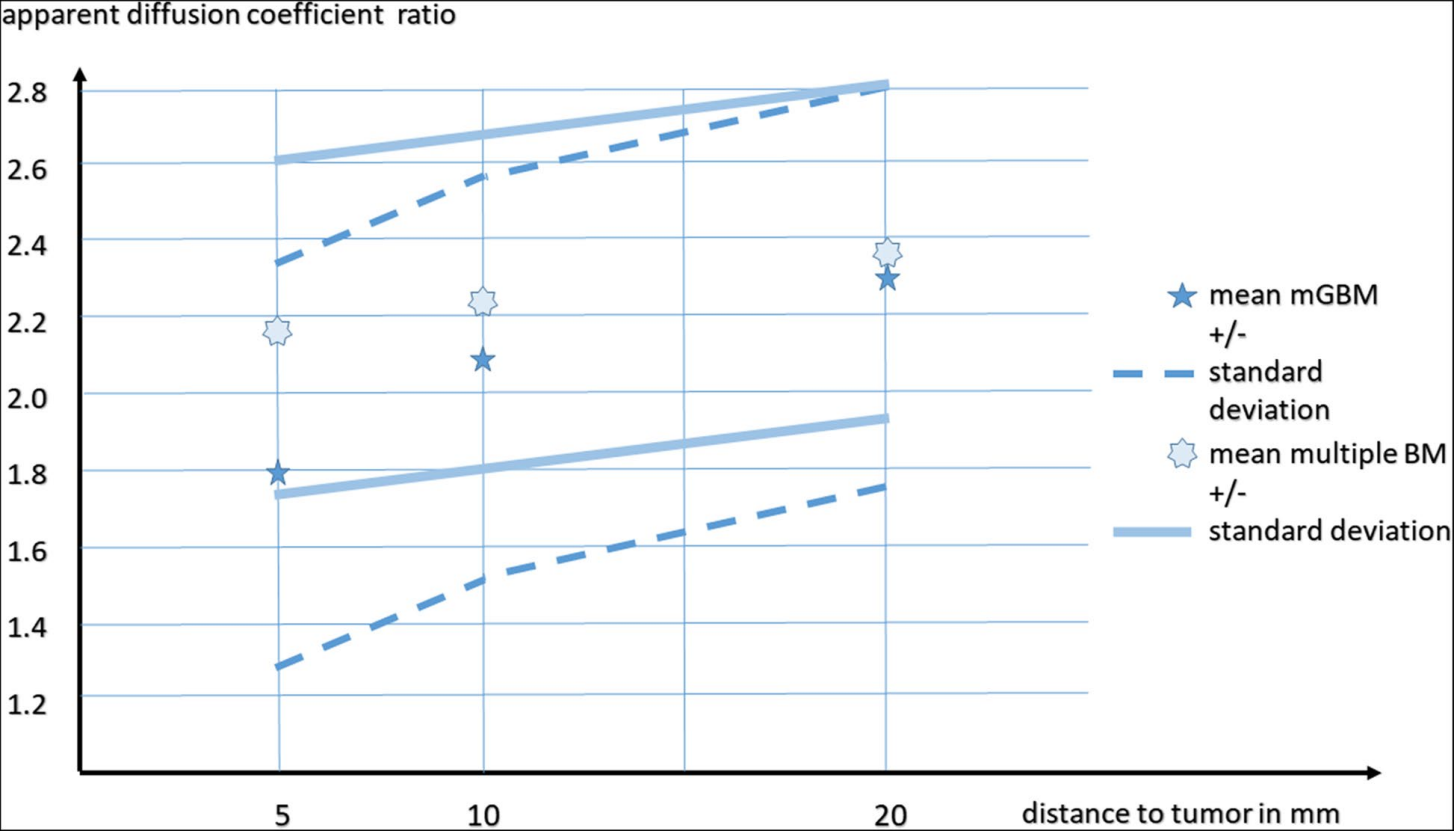
Gradient of the ADC ratios of the edema of glioblastoma with multiple foci and multiple brain metastases. ADC – apparent diffusion coefficient, mGBM – glioblastoma with multiple loci at presentation; BM – multiple brain metastasis

The AUC in ROC-analysis of ADC ratio (5 mm) for differentiation of GBM with multiple tumor lesions from multiple brain metastases was 0.72 with an optimized threshold of 1.86 (Youden's J, sensitivity 81%, specificity 60%). The ADC ratios are illustrated in Fig. 4.

### Interrater agreement

Inter rater agreement for measuring the ADC ratio of tumor was good with an ICC estimate of 0.87 and a 95% confidence interval of 0.73 to 0.94, respectively moderate for the measurement of the edemas (0.85; 0.72–0.92). Similar results with a minimal poorer agreement in perifocal edema were also shown in the measured T2-/FLAIR values.

**Fig. 5:**
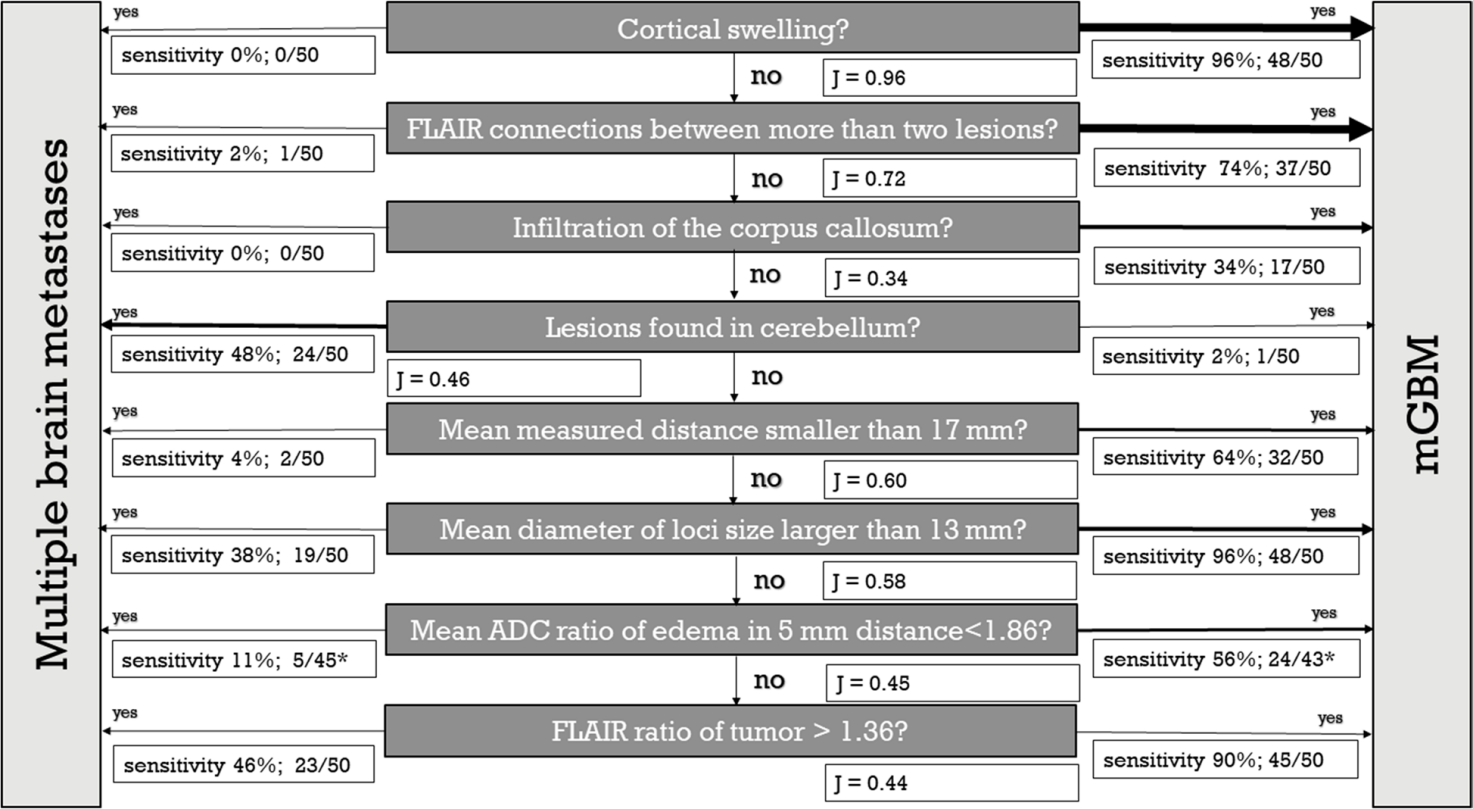
Decision flow chart. The sensitivity on the left equals (1-specificity) of the right site and vice versa. mGBM – glioblastoma with multiple foci; FLAIR – Fluid attenuated inversion recovery; ADC – apparent diffusion coefficient; J – Youden’s J; *-less than 50 patients with measurements due to the absence of edema

Based on the probability distribution of our cohort and AUC of ROC-analyses and Youden`s J analyses, a decision flow chart was created, which is shown in Fig. 5. Anatomical key structures were given higher ratings in some cases due to their high specificity.

## Discussion

In this observational study, a comprehensive imaging analysis involving 50 GBM patients with multiple tumor lesions and 50 patients with multiple brain metastases, respectively, revealed significant radiological differences between the two pathologies concerning tumor size, distance between lesions, ADC ratio of the nearest edema rim, within 5 mm surrounding the tumor lesion, and FLAIR-intensity-values of the tumor, as well as concerning the preference for distinct tumor locations such as corpus callosum. On the contrary, no significant differences were found for FLAIR-intensity-values or the size of the edema and tumor-edema-ratio. The most important morphological characteristic was cortical swelling, noticed in high-resolution T2-weighted sequences (better than in FLAIR). The FLAIR and ADC ratios were applied as a medium-term aid since unlikely T1- and T2-weighted imaging mapping, no high-resolution mapping procedures exist for FLAIR and DWI [21, 22].

It has been proven that MRI attributes of solitary brain tumors differs significantly from metastases regarding radiological features [23], MRI mass effect, midline-shift and edema [24], as well as morphology [25, 26]. Nevertheless, the problem appears to be much more complicated with multifocal or multicentric masses. The morphology and MR-attributes of brain metastases differs depending on the primary tumor [27], e.g. demonstrated for the ADC values of brain metastases of small cell lung cancer [17]. Glioblastoma with multiple foci at presentation have been classically divided in two entities: multifocal and multicentric mGBM [8]. In contrast to the literature, our cohort of GBM with multiple tumor lesions consisted of 34% multifocal and 24% multicentric GBM tumor lesions, as well as a never described 42% mixed type [9]. Based on the methodology of other studies, we assume that the mixed type was always counted among the multifocal GBM tumor lesions, which would then account for 76% of GBM patients with multiple tumor lesions in our study.

In a study from Iran, the ADC values/ratios of GBM, metastases and of edemas did not differ significantly [28]. This is largely consistent with our results, in which we did not detect any differences if the ADC values were measured within the area of 1 cm and 2 cm away from the tumor edge. But, when ADC was measured in the area directly adjacent to the tumor within 5 mm away from the tumor edge, a significant difference in ADC values was detected. A possible explanation for this finding may be a higher fluid content in this area in case of brain metastases. On the other hand, this could be an indicator of tumor cell infiltration (micro-invasion) in case of GBM. This is in line with the theoretical assumption that in GBM patients with multiple lesions the peritumoral edema is already microscopically infiltrated by tumor cells, and thus exhibiting a restricted water diffusion compared to the vasogenic edema of brain metastases.

On the contrary, no difference was found concerning the measured FLAIR-intensity-values, as previously shown in solitaire brain tumors [29]. Another MRI-approach to establish radiological distinguishing parameters for GBM with multiple lesions from multiple brain metastases may be the additional consideration of MR spectroscopy [27], MR perfusion [30] or susceptibility weighted imaging (SWI) [31, 32]. Since these additional imaging was not available in our patient cohort, it was not possible to include such parameters into the analysis of this study. Furthermore, multiparametric evaluation and [33] artificial intelligence [34] is also expected to play an important role in the future.

GBM is rarely located within the cerebellum and can be often misdiagnosed [35]. The coincidence of a cerebellar lesion location and mGBM seems to be even rarer. On the other hand, GBM can be predominantly located within the corpus callosum, which seems to be a rare location for brain metastases. A few case reports about metastasis in the corpus callosum exist [36, 37], but since most metastases appear to spread to the brain via the bloodstream, the corpus callosum is omitted in larger studies [38, 39].

### Limitations of the study

Main limitations of the study are its retrospective nature and the potential for selection bias. Incorporating a more diverse patient population or multicenter data could enhance the generalizability of the findings. A subset of aggressive cancers (e.g., testicular cancer) do not appear in our dataset.

Since the morphology and configuration of GBM with multiple lesions differ from GBM with a singular manifestation, we did not make a comparison for typical patterns, like the “Pseudopalisade”-sign [40]. A purely visual evaluation and manual measurements were carried out. Automatic volumetry was not used because today's segmentation algorithms for cerebral masses are very prone to errors. While there were no significant differences in tumor and edema diameter as well as tumor-edema-ratio in our study, a volumetric study in solitary tumors has already shown a larger mean edema volume in relation to the mean tumor volume in brain metastases compared with GBM [41].

Future studies might explore the potential of advanced MRI techniques or artificial intelligence in further refining the diagnostic process. As mentioned above, the use of normalized mapping methods could be more precise than the use of ratios and may reveal more differences in the future.

## Conclusion

Statistically, GBM with multiple tumor lesions and multiple brain metastases can be distinguished very well, especially by FLAIR connections and anatomic landmarks. In individual cases with multicentric GBM, however, the radiological differentiation from multiple brain metastases may not be possible. The consideration of multiple radiological parameters such as distance between loci, ADC of tumor near edema, and tumor size may be supportive to distinguish between these two differential diagnoses. Because of the amount of these parameters and the complexity of distribution an experienced neuroradiologist is required for a safe diagnosis. This differentiation could be an exciting and challenging playing field for neural networks and AI in the future.

## Data Availability

The datasets used and analyzed during the current study are available from the corresponding author on reasonable request.
